# Outcomes of Fat Grafting in the Active Versus Quiescent Phase of Localized Scleroderma

**DOI:** 10.1177/22925503231167444

**Published:** 2023-04-17

**Authors:** Anna Wang, Lisanne Grünherz, Ilaria V. De Martini, Mauro Vasella, Pietro Giovanoli, Nicole Lindenblatt

**Affiliations:** 1 Department of Plastic Surgery and Hand Surgery, 27243 University Hospital Zurich, Zurich, Switzerland; 2 Institute of Diagnostic and Interventional Radiology, 27243 University Hospital Zurich, Zurich, Switzerland

**Keywords:** active disease, fat grafting, linear scleroderma, progressive hemifacial atrophy, greffe de matière grasse, hémiatrophie faciale progressive, maladie active, sclérodermie linéaire

## Abstract

**Introduction:** Progressive hemifacial atrophy (PHA) and linear scleroderma (LS) are both rare conditions and defined by atrophy and/or sclerosis of the skin and subcutaneous tissue. The ideal timing of reconstructive intervention in these patients is controversial. We compared the outcome and satisfaction of autologous lipofilling performed during active and stable phases of the diseases in adults. **Methods:** A retrospective chart review was conducted with all patients diagnosed with PHA or LS between 2007 and 2019 in our department. We analysed demographic data, clinical features, and surgical procedures. The changes in symmetry, volume and skin texture were rated by surgeons at 1 week, 3 months and 6 months compared to the preoperative presentation. We compared the outcomes of patients treated during the active and the stable phase of the disease. Additionally, patients were asked to fill out a quality-of-life questionnaire. **Results:** We found a total of 11 patients diagnosed with PHA and LS, 8 of whom had undergone autologous fat injections to correct facial asymmetry. Of those, 4 patients were treated in their active and 4 in their stable phase. We found similar treatment outcomes in both groups. The social component had the greatest negative effect on patient's quality of life. **Conclusion:** In this small cohort, autologous fat grafting during the active phase did not appear to be inferior to fat grafting during the stable phase. It could be a safe technique for correction of PHA and LS during the active phase of disease.

## Résumé

**Introduction:** L’hémiatrophie faciale progressive (HFP) et la sclérodermie linéaire (SL) sont toutes deux des affections rares définies par l’atrophie ou la sclérose de la peau et des tissus sous-cutanés. Le moment idéal pour procéder à une intervention reconstructive chez ces patients. Les auteurs comparent les résultats et la satisfaction associés à un lipofilling autologue effectué pendant les phases active et stable de la maladie chez les adultes. **Méthodologie:** Les auteurs ont procédé à une analyse rétrospective des dossiers de tous les patients ayant obtenu un diagnostic d’HFP ou de SL entre 2007 et 2019 au sein de leur département. Ils ont analysé les données démographiques, les caractéristiques cliniques et les interventions chirurgicales. Les chirurgiens ont évalué les changements de symétrie, du volume et de la texture de la peau par rapport à la présentation préopératoire au bout d’une semaine, de trois mois et de six mois. Les auteurs ont comparé les résultats chez les patients traités pendant les phases active et stable de la maladie. De plus, les patients ont été invités à remplir un questionnaire sur leur qualité de vie. **Résultats:** Les auteurs ont recensé un total de onze patients ayant un diagnostic d’HFP ou de SL, dont huit ont subi des injections de graisse autologues pour corriger une asymétrie faciale. De ce nombre, quatre ont été traités pendant la phase active et quatre, pendant la phase stable. Les auteurs ont observé des résultats thérapeutiques semblables dans les deux groupes. L’aspect social avait l’incidence la plus négative sur la qualité de vie des patients. **Conclusion:** Dans cette petite cohorte, la greffe de graisse autologue pendant la phase active ne semblait pas donner de moins bons résultats que la greffe de graisse pendant la phase stable. Cette technique pourrait être sécuritaire pour corriger une HFP ou une SL pendant la phase active de la maladie.

## Introduction

Progressive hemifacial atrophy (PHA) is a rare disease with typically hemifacial atrophy of unknown etiology.^1^ Its precise incidence is unknown due to a lack of standardized diagnostic criteria and overlapping features of PHA and linear scleroderma (LS) *en coup de sabre* (ECDS). It is estimated as 1-2 per million.^2^ The disease is characterised by a hemifacial atrophy of subcutaneous tissue, fat, muscle and osseocartilaginous structures inferior to the forehead, typically involving dermatomes of the trigeminal nerve. Patients have a sunken hemiface appearance. Epidermal involvement in PHA is minimal.^1^

LS is commonly termed ECDS and is the most common subtype of localized scleroderma, an autoimmune condition. Localized scleroderma typically presents in childhood with an annual incidence of 1 to 3 per 100 000 individuals. It is defined by sclerosis and resulting atrophy of the skin and subcutaneous tissues. Lesions are classified as circumscribed, linear, generalized, and pansclerotic according to the Padua criteria.^3^ LS generally occurs unilaterally in form of a blaschkoid distribution, potentially involving deeper neurologic, ocular or oro-dental tissues.^4^ Skin involvement is typical and is described as hyperpigmented, shiny, firm, and displaying alopecia.^1^

Current literature suggests that PHA and ECDS lie on a spectrum of localized scleroderma.^4^ In total, 28% to 42% of patients have coexisting ECDS and PHA.^1^ Both diseases are considered as self-limiting. An initial active phase lasting 2 to 10 years is followed by a stable phase.^4^

The first-line treatment for localized scleroderma and its subtypes is conservative and based on steroids and conventional disease-modifying antirheumatic drugs, specifically methotrexate. Surgical interventions offer functional and aesthetic rehabilitation, most frequently by autologous fat grafting or microsurgical flaps.^4^ Facial reconstruction, in particular, may minimize psychosocial effects in both conditions.^5^

Regarding the ideal timing of reconstructive intervention controversy exists. While most authors propose proceeding after a 1 to 2 year hiatus of stable disease, a recent case study suggests that fat grafting may positively influence the disease in the active phase.^6^ Moreover, treatment at a younger age (<14 years) was correlated with higher overall satisfaction in a recent report.^5^

The fat grafting technique presents a particular challenge in patients with PHA and LS, as these patients have a higher resorption rate compared to nonaffected patients. Slack et al^7^ propose suboptimal blood supply in the recipient bed or a change in Romberg diseased tissue as possible reasons. When treated with corticosteroids, scleroderma patients have a significantly reduced number of stem cells in their fat, implying a possible impact on fat resorption.^8^ Other authors describe a slower fat resorption rate after additional injection of adipose derived stem cells.^9^ Due to the unpredictable fat resorption rates, current treatment regimens typically require numerous interventions, representing a burden both to patients and the health-care system. Some authors suggest initial volume overcorrection to improve the outcome.^10^ However, whether this leads to fewer interventions has not been analysed systematically.

The aim of our case series was to compare the physician's outcome satisfaction after fat grafting during the active and stable phases of PHA and LS.

## Methods

### Patients

We retrospectively researched medical records from 2007 to 2019 at the Department of Plastic and Hand Surgery. Patients with a clinical diagnosis of PHA and/or LS were identified. This study was approved by the Ethics Commission (Project ID 2019-01282). Patients gave written consent for the publication of patient information and full facial photographs.

Medical records were evaluated with respect to the following: type and severity of disease, gender, age at onset, preoperative therapies, affected side, skin texture changes, phase of the disease at the time of intervention (stable/active), patient age at surgical intervention, number of interventions, donor site, injection volume of microfat and nanofat and two dimensional (2D) photographs (preoperative and postoperative).

We defined active disease as an observed progression in a change of skin texture or asymmetry in the face during the year of intervention. In cases in which MR imaging was available, atrophy of subcutaneous fat, muscle and bone was evaluated by a radiologist in order to grade the severity of disease into mild, moderate or severe according to the grading scale developed by Raposo-Do-Amaral et al.^11^

### Surgical Technique

Surgeries were performed with the same technique. Fat was harvested from the abdomen and thighs after infiltration with a liposuction solution containing Ringer's lactate solution (500 ml), aqua (200 ml), adrenaline solution 1 mg/1 ml (1:1000)*.* A Tulip Infiltrator with 2.1 mm was used (Tulip^®^ GEMS Tumescent, Tulip Medical Products). A high-negative-pressure liposuction procedure was performed using a standard liposuction device. Fat was harvested with a multiport 2.4-mm cannula with sharp microports and 20 port configurations (Tulip^®^ GEMS Tonnard Harvester, Tulip Medical Products). After filtering, lipoaspirate is washed with saline to clear it from tissue remnants (microfat).^12^ Microfat was injected subcutaneously with a Tulip Injector 0.9 mm (Tulip^®^ GEMS Injector, Tulip Medical Products) into the areas with volume loss.

For the nanofat, lipoaspirate was mechanically emulsified after rinsing with saline. Emulsification of the fat was achieved by shifting the fat between two 1-ml syringes with Luer-Lok (BD Luer-Lok 1-ml syringe, Becton Dickinson) connected by a female-to-female Luer-Lok connector. First between 1.4 mm Luer-Lok and secondly between 1.2 mm Luer-Lok. After 30 passes, the fat changed into an emulsion and took on a whitish appearance. Final filtration occurs through a Nano Transfer Cartridge (Tulip^®^ Nanofat Transfer, Tulip Medical Products). This effluent was injected with the Tulip Injector (0.9 mm) intradermally into the skin lesions with either hyperpigmentation or alopecia. Slight volume overcorrection was targeted.

### Outcome Assessment—2D Photograph Analysis and Quality of Life

Satisfaction regarding change in the symmetry, volume, and skin texture was rated by surgeons by comparing preoperative and postoperative 2D photographs. All surgeons rated the cosmetic results on a 5-point scale with the following definitions: 1  =  dissatisfied, no improvement; 2  =  partially satisfied; 3  =  half satisfied, 4  =  mostly satisfied; and 5  =  totally satisfied.^5^ This 5-point scale was used by Slack et al after reconstruction in PHA. We chose to use the same scale to facilitate comparison. All photographs were rated by 4 plastic surgeons from our department of Plastic and Hand Surgery.

A questionnaire aiming at measuring disease-specific impairment of quality of life was sent to consenting patients months to years postoperatively. This questionnaire from Palmero et al^13^ was used, as it was specifically developed to measure quality of life in this patient population. The questionnaire was patterned after 3 validated instruments: the Skin Cancer Index, the Facial Plastic Surgery Outcomes Evaluation, and the Nasal Obstructive Symptoms Evaluation scale. Consisting of 13 questions it covers 4 domains (appearance, social, emotional, and symptoms) with a 5-point response format, 1 representing “very much” to 5 “not at all.” Scores were obtained and computed as previously described. Higher scores indicate patient satisfaction with less impairment of the quality of life.

### Statistical Analysis

Statistical analysis was performed using open-source software (R Version 3.62) and Data visualization with dplyr (v. 1.0.0) and ggplot2 (v. 3.3.2) packages. An unpaired 2-sided *t*-test was used to determine any significant differences between the 2 comparative groups of patients. To compare physician's satisfaction between patients treated during active and stable phases, a Mann-Whitney-Wilcoxon test was performed. A *P*-value of < .05 was defined as significant. We provide p-values to aid interpretation but due to the limited number of patients, we consider this analysis to be exploratory.

## Results

A total of 11 patients with PHA or LS, treated between 2007 and 2019 in our Department of Plastic and Hand Surgery are included in our case series. Of those, 9 were female and 2 were male (F:M ratio  =  4.5:1), with a median age of 19 years (interquartile range[IQR] 13-27) at onset of the disease. Six patients were diagnosed with LS and 5 patients with PHA. The affected side of the face was left (n  =  6) and right (n  =  5). The severity of disease was mild in 4, moderate in 2, and severe in 2 patients. In addition, 3 patients were categorized as mild, as no MRI was available and atrophy of subcutaneous tissue was clinically visible, but atrophy of muscle or bone was not proven.

### Surgery

**Figure 1.**
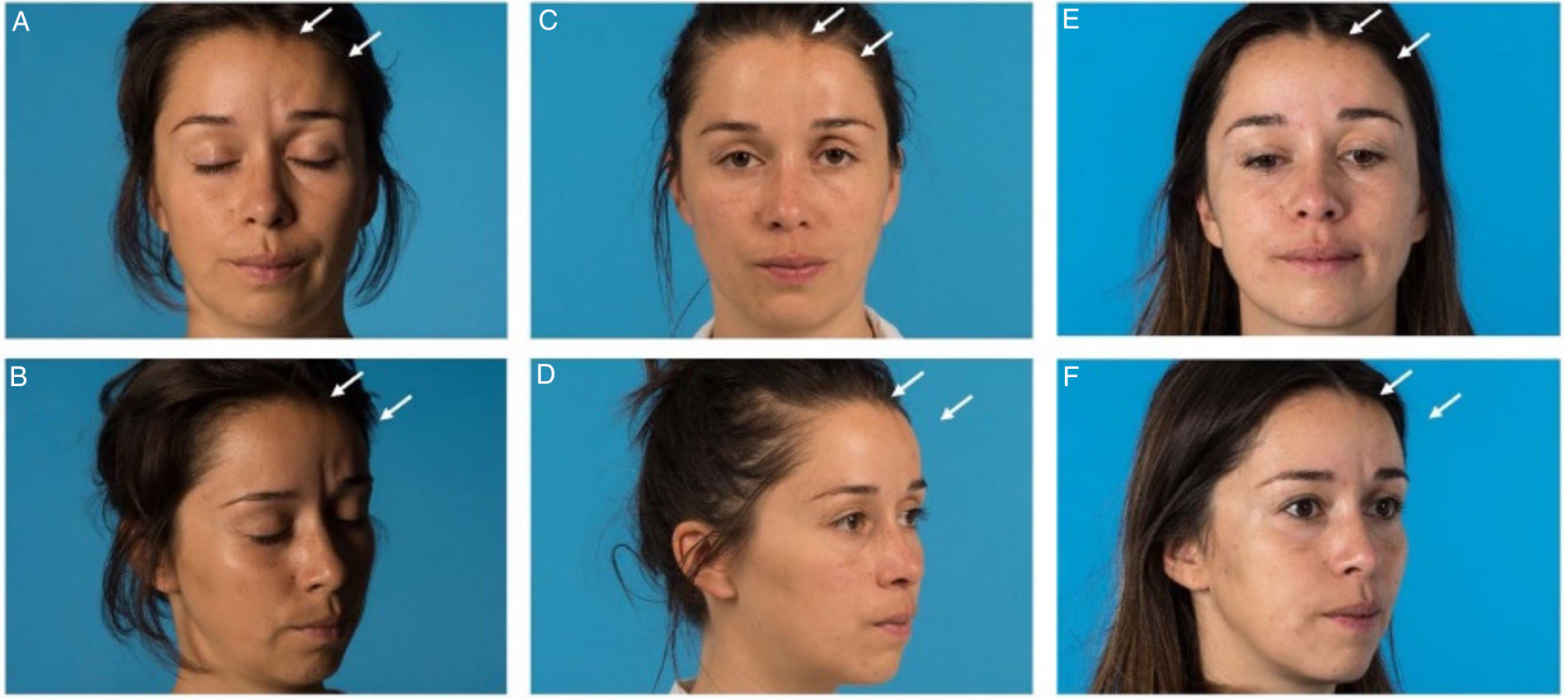
Patient diagnosed with active linear scleroderma (LS) was treated with fat grafting twice. Microfat was used for volume correction at the frontal and glabella region and nanofat was used for the correction of hyperpigmentation in the frontal region (hairline). (**A** and B): front and oblique lateral view of preoperative face. (**C** and D): front and oblique lateral view of the face 3 months after the first surgery. At this point the postoperative change was rated as follows, given in median (range): symmetry 4 (3-4), volume 3.5 (2-4) and skin change 4 (2-4). (E and F): front and oblique lateral view of the face 3 months after the second surgery. At this point the postoperative change was rated as follows: symmetry 5 (4-5), volume 5 (3-5), and skin change 3.5 (3-4).

**Figure 2.**
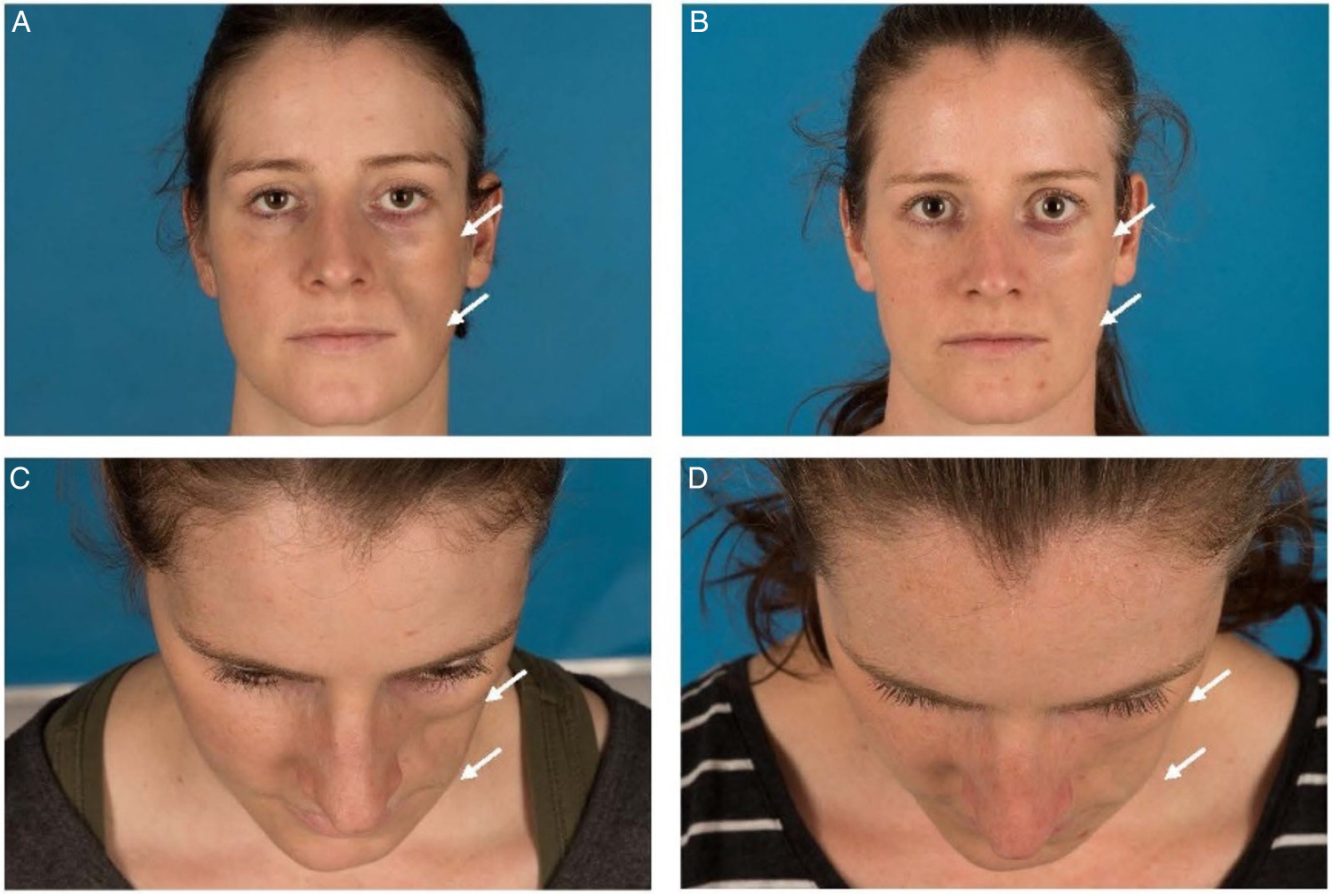
Patient diagnosed with active progressive hemifacial atrophy (PHA) treated with fat grafting. Microfat was used for volume correction at the cheek and nanofat was used for the infraorbital region. (A and B): Front and cranial view of preoperative face. (C and D): Front and cranial view of the face 6 months after the first surgery. At this point the postoperative change was rated as follows, given in median (range): Symmetry 3.5 (3-5), volume 4 (3-5), and skin change 3.5 (2-4).

Eight patients received surgery with combined lipofilling (microfat and nanofat) to correct facial deformities (Table 1, Figures 1 and 2). Three patients did not undergo surgery after consultation. The median age at surgery was 31.5 years (IQR 25-34.8). Four patients were treated during the stable phase of their disease and 4 in the active phase of their disease. The median number of interventions was 1 (IQR 1-1.25). In the following we described 2 patients receiving multiple fat grafting sessions.

**Table 1.** Characteristics of Patients who Received Surgery Between 2007 and 2019 with Lipofilling to Correct Facial Deformities

Active phase at intervention	Age at surgery (yrs)	Sex	Diagnosis	Severity of disease (mild/moderate/severe)	Follow up (months)	Number of interventions	Total injected volume of microfat (ml)	Nanofat used to improve hyperpigmentation
Patient 1	33	F	LS	moderate	3	2	2	Yes
Patient 2	53	F	PHA	mild	6	1	7	Yes
Patient 3	28	F	LS	mild	3	1	14	Yes
Patient 4	31	F	PHA	moderate	6	1	33	Yes
Stable phase at intervention								
Patient 5	15	M	LS	mild	3	1	4	No
Patient 6	16	F	LS	mild	3	1	11	No
Patient 7	40	M	PHA	mild	6	1	3.2	No
Patient 8	32	F	PHA	severe	3	3	32	No

Abbreviations: LS, linear scleroderma PHA, progressive hemifacial atrophy; F, female; M, male.

The first patient was a 32-year-old female with PHA in a stable phase, suffering from atrophy of the whole right-sided face, especially, at the zygomatic, malar, temporal, and mandibular region. The right upper lid showed a volume deficit with a resulting A-frame deformity.

The first surgery was performed with microfat grafting (32 ml) of the right-sided face, correcting all the above-mentioned regions. The patient was very satisfied 3 months postoperatively, because the lack of volume on her face have mostly been corrected. Small volume deficits remained at the upper face (paramedian forehead, malar and temporal region). The second session took place 4.5 months after the first session. This time 22 ml was injected especially to the upper face. Only the right-sided forehead showed a modest volume deficit, 6 months after the second session. Finally, the patient requested additional aesthetical corrections to the glabella, the malar region and a correction of her A-frame deformity. For the forehead, she opted for a reconstruction with a titan mesh over fat grafting, as it seemed, that fat resorption rate was higher on the forehead. Microfat (4 ml) was injected in her third session to glabella, the malar region and to correct the A-frame deformity. The patient was completely satisfied 3 months after the third session with the contour and volume of her face. Consent for the publication of clinical photographs was not provided.

The second patient was a 37-year-old female suffering from active LS on the left-sided forehead and the glabella region with volume deficit and hyperpigmentation. During the first surgery 2 ml microfat was injected to the above-mentioned areas and the hyperpigmented area was injected with 0.3 ml nanofat. The patient reported that she was most satisfied with the results 1 week postoperatively, as fat resorption increased afterwards. The second session was done 1.5 years after the first session with 2.3 ml of microfat and 0.4 ml of nanofat injected at the same sides. The patient was satisfied 3 months postoperatively and not disturbed by her asymmetric appearance anymore, with very little atrophy left.

Patients in our cohort had multiple recipient sites containing the frontal region (n  =  5), cheek (n  =  5), the mental region (n  =  2), the periorbital region (n  =  3), and the nose (n  =  2). The donor site consisted of the abdomen (n  =  6) or thighs (n  =  2). The median injection volume of the microfat subcutaneously was 9 ml (IQR 3.8 - 15.3 mL) and that of nanofat intradermally was 0.3 mL (IQR 0-2.1 ml). None of the patients had any complications at either the donor or recipient site during follow-up.

### Outcome Assessment—2D Photograph Analysis and Quality of Life

**Figure 3.**
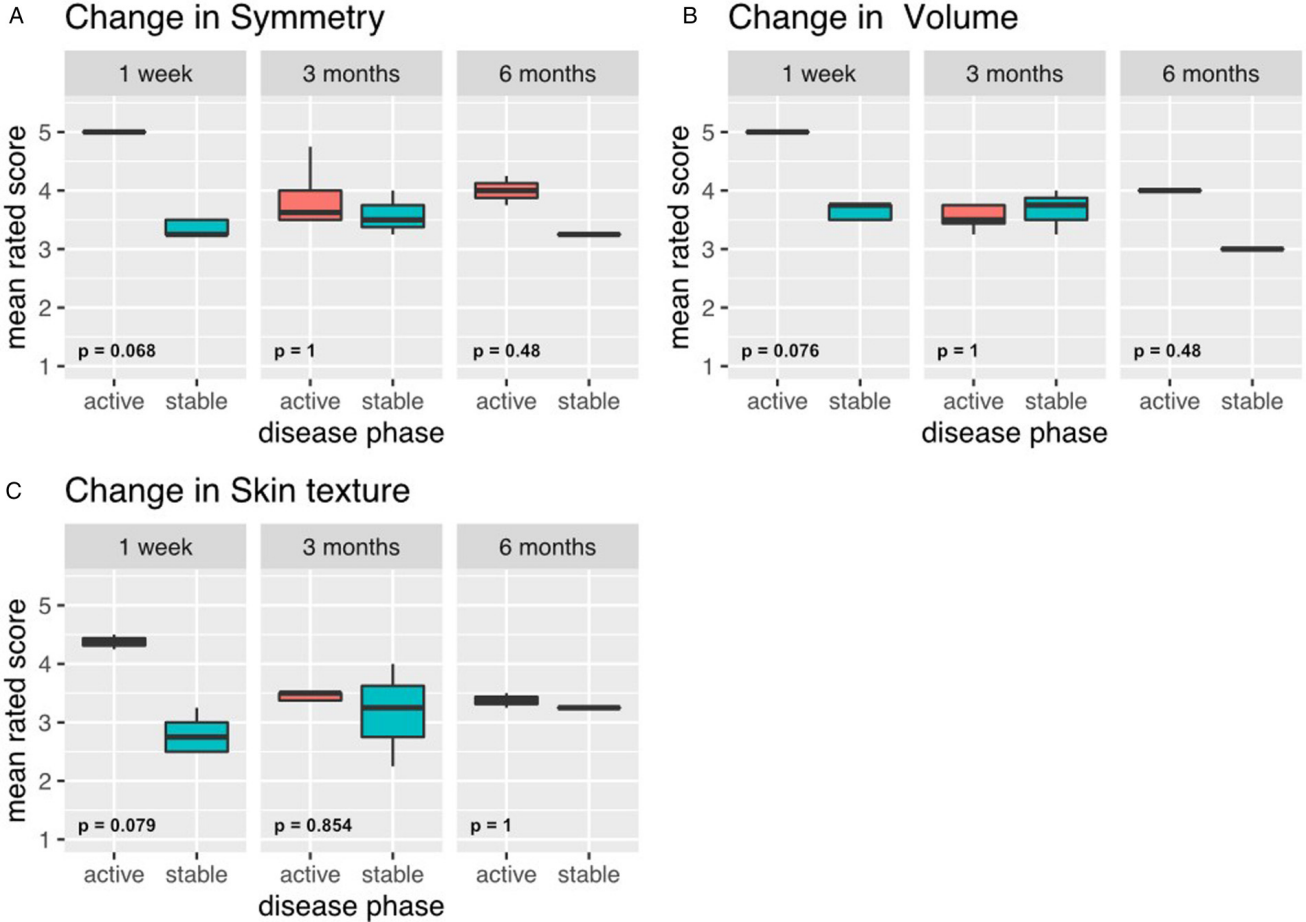
Comparison of physician's postoperative outcome satisfaction, by disease phase at the time of intervention. (A) Satisfaction with change in symmetry 1 week, 3 months, and 6 months postoperatively. (B) Satisfaction with change in volume 1 week, 3 months and 6 months postoperatively. (C) Satisfaction with change in skin texture 1 week, 3 months and 6 months postoperatively.

There was a tendency towards better physician-rated outcome satisfaction in patients treated during the active phase in all 3 rated modalities (change in symmetry *P*  =  .068, change in volume *P*  =  .076, change in skin texture change *P*  =  .079), especially at first week post intervention (Figure 3). Ratings at 3 months postintervention were similar between both groups. The change in symmetry and volume in the group treated in the active phase was slightly higher 6 months postoperatively, whereas the change in skin texture stayed similar between both groups (Table 2 and Figure 3). Satisfaction for patients treated during the active phase was similar to or better compared to patients treated during the stable phase across all time points and all rated modalities. The severity of the disease and the preoperative asymmetry did not appear to be associated with postoperative change in symmetry (data not shown).

**Table 2.** Physician's Postoperative Outcome Satisfaction, by Disease Phase.

	Group treated in active phase	Group treated in stable phase
Change in symmetry		
1 week	5 (5-5)	4 (1-5)
3 months	4 (3-4)	3.5 (2-5)
6 months	4 (3-5)	3 (3-4)
Change in volume		
1 week	5 (5-5)	4 (1-5)
3 months	4 (2-4)	3.5 (3-5)
6 months	4 (2-5)	3 (2-4)
Change in skin texture		
1 week	4.5 (4-5)	3 (1-4)
3 months	4 (1-4)	3.5 (1-4)
6 months	3.5 (2-4)	3.5 (2-4)

Change in symmetry, volume, and skin texture were evaluated through a 5-point scale used by Sleck et al.^5^ changes were assessed compared to the preoperative presentation. data are given as median (range). Timepoints are time postsurgery

### Quality of Life

5 out of 8 patients completed the survey. Results are summarized in Table 3. All responders had been unhappy with their appearance and/or their skin changes prior to surgery, prompting them to undergo surgical intervention. In addition, 2 patients stated loss of confidence, and another patient stated both headache and eye pain as well as the wish for a halt of the disease as motivations for surgery. One patient stated additional loss of hair as a motivation for surgery.

**Table 3.** Quality-of-Life Survey Data, by Disease Phase.

Question	Quality of life factor	Group treated in active phase		Group treated in stable phase	
		Unstandardized raw score (1-5)	Standardized score (0-100)	Unstandardized raw score (1-5)	Standardized score (0-100)
Appearance subscale		**1.8** (**1-2.5)**	**18.8** (**0-37.5)**	**2 (–)**	**25 (–)**
**1**	Worried about appearance	2 (1-5)	25 (0-100)	2 (–)	25 (–)
**2**	Concerned about how the disease affects attractiveness	2.5 (1-5)	37.5 (0-100)	2 (–)	25 (–)
**3**	Concerned about how appearance can improve	1.0 (1-5)	0 (0-100)	2 (–)	25 (–)
**4**	Considered surgery to alter appearance	1.5 (1-5)	12.5 (0-100)	3 (–)	50 (–)
Social subscale		** 1.0** (**1)**	** **(**0) (–)**	**1** (–)	**0** (–)
**5**	Uncomfortable meeting new people	1.0 (1-5)	0 (0-100)	1 (–)	0 (–)
**6**	Bothered by people's stares	1.0 (1-5)	0 (0-100)	1 (–)	0 (–)
**7**	Avoided social activity	1.0 (1-5)	0 (0-100)	1 (–)	0 (–)
Emotional subscale		**3.0** (**2.5-4)**	**50** (**37.5-75)**	**3** (–)	**50** (–)
**8**	Felt anxious about condition	4 (2-4)	75 (25-75)	2 (–)	25 (–)
**9**	Felt frustrated about condition	2.5 (1-5)	37.5 (0-100)	3 (–)	50 (–)
**10**	Worried about progression of asymmetry	3 (1-4)	50 (0-75)	3 (–)	50 (–)
Symptoms subscale		**3** (**4-5)**	**50** (**50-100)**	**1** (–)	**0**(–)
**11**	Concerned about skin changes	3 (2-4)	50 (25-75)	3 (–)	50 (–)
**12**	Bothered by headaches or eye problems	3 (1-5)	50 (0-100)	1 (–)	0 (–)
**13**	Bothered by surgical complications	5 (1-5)	100 (0-100)	1 (–)	0 (–)
Total score		**2.5**	**37.5**	**2.0**	**25**

A 5-point response format was used to assess 4 quality of life domains (appearance, social, emotional, and symptoms). total score and standardized scores were computed as previously described in Palmero et al.^13^ data are given as median (range). As only 1 patient who was treated in the stable phase answered the questionnaire, the range is omitted in this case. Subscales are in bold font.

In the group treated in the active phase, the social subscale demonstrated the lowest standardized scores and possibly the greatest negative effect on the patient's quality of life compared to the other subscales. The symptoms subscale demonstrated the highest standardized scores. The patient treated in the stable phase of disease also showed the lowest standardized score in the social subscale. However, the standardized score of the emotional subscale was the highest.

Four out of 5 patients considered additional surgery in the future and would recommend surgical intervention to other patients with both diseases. Overall satisfaction with the surgery varied from extremely satisfied (n  =  1), very satisfied (n  =  2), and somewhat satisfied (n  =  1) to not at all satisfied (n  =  1).

## Discussion

Our surgical cohort demonstrates the feasibility of combined lipofilling (microfat, nanofat) in both the active and stable disease phases of patients with PHA and LS. However, fat resorption remains a challenge, leading to potentially decreased long-term outcome satisfaction.

The characteristics of our patient collective are like those described in the literature, with a higher prevalence of females and an average onset of disease in the first 2 decades of life. Clinical and histopathologic features were also similar.^1^ Recipient and donor sites were similar to those previously described,^10^ however patients in our collective received a lower number of treatments and a lower average number of injections. As treatment at younger ages becomes more and more important because it showed higher satisfaction, it is crucial to know whether treatment during an active phase of disease is safe.

To the best of our knowledge, this is the first case series evaluating the experience and satisfaction in a patient collective receiving fat grafting during the active course of disease compared to interventions in a stable phase. In the reported literature, most interventions are done after stabilization of disease partially based on evidence that early reconstruction does not alter the course of disease.^5^ A literature review of Rodby et al^10^ from 2016 cited 147 PHA cases. Of these, 28 cases were treated at a younger age, including 2 studies from Hunstad et al and Slack et al. Hunstad et al^6^ described 1 patient with fat grafting during the active phase, in whom remission of the disease was observed during a 14-year follow-up. Slack et al^5^ reported treatments with low complication rates in a patient population under 14 years. However, this study did not explicitly specify whether patients were in the active phase of the disease during their treatment.

In the case series presented here, the surgeon-rated outcome satisfaction in both groups, was highest in the early postoperative period. The level of satisfaction descended during the third and 6 months postoperatively. This may be due to the rate of fat resorption in this specific patient population.^7, 8^

The group treated in the active phase had similar to slightly favorable outcomes. This might be due to different rates of fat resorption between both groups. Skin texture change demonstrated less improvement compared to the change in symmetry and volume, indicating that an additional procedure might be necessary to correct hyperpigmentation to facilitate better outcomes and higher satisfaction.

Disease-related quality of life is an important outcome measure to facilitate patient-centric care. Overall patient satisfaction with the surgery varied, but most of the patients reported to be satisfied with their appearance after the surgery, considered further surgery in the future and considered recommending the intervention to other patients. This shows that fat grafting has helped our patients in improving the appearance of their face. In contrast to Palmero et al,^13^ our surgical cohort showed lowest scores in the social subscale, in both groups. Patients treated in our clinic felt uncomfortable meeting new people and avoided social interactions rather than being bothered by the appearance of the disease. The fact that symptoms and emotional impact did not appear to be the centre of attention for our patients might explain why most patients underwent 1 single session of fat grafting. They might not have the motivation, strength, time or funds required to undergo frequent treatments. The subscale with the highest scores was the symptoms subscale in the group treated during the active phase, corresponding to the results of Palmero et al,^13^ emphasizing a low impact of the disease on physical and functional deficits and perceived complications of surgery. In the patients treated during the stable phase, the emotional subscale showed the highest score, implying that the disease had a comparatively low impact on anxiety, frustration and concerns about the condition.

Limitations of this surgical case series include its limited sample size and retrospective nature. Results of the quality-of-life questionnaire should be interpreted cautiously, as these had to in some cases be obtained months to years after the surgical intervention. Prospective and controlled studies would be of interest, however, might be challenging to achieve given the rarity of the disease.

In conclusion, autologous fat grafting during the active phase did not appear to be inferior to fat grafting during the stable phase in this small clinical case series. To understand challenges concerning fat resorption, further research is needed to determine whether the fat quality of this special patient population plays a significant role.
